# Prophylactic Administration with Methylene Blue Improves Hemodynamic Stabilization During Obstructive Jaundice–Related Diseases’ Operation: a Blinded Randomized Controlled Trial

**DOI:** 10.1007/s11605-022-05499-3

**Published:** 2023-04-26

**Authors:** Jian Huang, Xian Gao, Moran Wang, Zhen Yang, Lunli Xiang, Yongshuai Li, Bin Yi, Jianteng Gu, Jing Wen, Kaizhi Lu, Hongwen Zhao, Daqing Ma, Li Chen, Jiaolin Ning

**Affiliations:** 1 Department of Anesthesiology, Southwest Hospital, Third Military Medical University, 30 Gaotanyan Road, Chongqing, 400038 China; 2 Department of Nephrology, Southwest Hospital, Third Military Medical University, 30 Gaotanyan Road, Chongqing, 400038 China; 3 Division of Anaesthetics, Pain Medicine and Intensive Care, Department of Surgery and Cancer, Faculty of Medicine, Imperial College London, Chelsea and Westminster Hospital, 369 Fulham Road, London, SW109NH UK; 4 Breast Disease Center, Southwest Hospital, Third Military Medical University, Chongqing, 400038 China

**Keywords:** Obstructive jaundice, Systemic vascular resistance, Hemodynamics, Methylene blue

## Abstract

**Objectives:**

Patients with obstruction jaundice are at a high risk of hypotension and need high volume of fluids and a high dose of catecholamine to maintain organ perfusion during operation procedure. All these likely contribute to high perioperative morbidity and mortality. The aim of the study is to evaluate the effects of methylene blue on the hemodynamics in patients undergoing surgeries associated with obstructive jaundice.

**Design:**

A prospective, randomized, and controlled clinical study.

**Setting:**

The enrolled patients randomly received 2 mg/kg of methylene blue in saline or saline (50 ml) before anesthesia induction. The primary outcome was the frequency and dose of noradrenaline administration to maintain mean arterial blood pressure over 65 mmHg or > 80% of baseline, and systemic vascular resistance (SVR) over 800 dyne/s/cm^5^ during operation. The secondary outcomes were liver and kidney functions, and ICU stay.

**Patients:**

Seventy patients were enrolled in the study and randomly assigned to receive either methylene blue or control (*n* = 35/group).

**Results:**

Fewer patients received noradrenaline in the methylene blue group when compared with the control group (13/35 *vs* 23/35, *P* = 0.017), and the noradrenaline dose administrated during operation was reduced in the methylene blue group when compared with the control group (0.32 ± 0.57 mg vs 1.787 ± 3.51 mg, *P* = 0.018). The blood level of creatinine, glutamic oxalacetic transaminase, and glutamic–pyruvic transaminase after the operation was reduced in the methylene blue group when compared with the control group.

**Conclusions:**

Prophylactic administration of methylene blue before operation associated with obstructive jaundice improves hemodynamic stability and short-term prognosis.

**Question:**

Methylene blue use prevented refractory hypotension during cardiac surgery, sepsis, or anaphylactic shock. It is still unknown that methylene blue on the vascular hypo-tone associated with obstructive jaundice.

**Findings:**

Prophylactic administration with methylene blue improved peri-operative hemodynamic stability, and hepatic and kidney function on the patients with obstructive jaundice.

**Meanings:**

Methylene blue is a promising and recommended drug for the patients undergoing the surgeries of relief obstructive jaundice during peri-operation management.

**Supplementary Information:**

The online version contains supplementary material available at 10.1007/s11605-022-05499-3.

## Background

Obstructive jaundice is induced by the common bile duct being blocked completely or partially, and then the bile overflows from the digestive system into the circulation, which will lead to all vital organ damage.^1–5^ Surgical interventions to relieve obstructive jaundice are essential but are often associated with a high postoperative morbidity and mortality. It has been reported that improved preoperative preparation and peri-operation management may greatly contribute to a favorable outcome, but those are not optimized yet. Patients with obstructive jaundice often presented to be hypotensive after anesthesia induction and are at a high risk of severe hypotension. Thus, the patients often need a large volume of fluid loading and a large dose of vasopressors to maintain adequate blood pressure.^6,7^ Methylene blue (MB), as a potent inhibitor of nitric oxide (NO) synthase, has been successfully used to combat refractory hypotension associated with cardiopulmonary bypass, septic shock, liver transplantation, and anaphylactic shock.^8–10^ Methylene blue is the second-line drug to treat this refractory hypotension; however, it was found that methylene blue improved the hemodynamics in the critical ill patients with refractory hypotension, and the patients may benefit more from early than later administration.^11^ To date, it remains unknown whether the patients with obstructive jaundice are also benefited from prophylactic administration of methylene blue. The patients, who were scheduled to undergo surgeries for relieving obstructive jaundice, were enrolled to evaluate the effects of prophylactic administration of methylene blue on the hemodynamic status and organs’ function in this trial.

## Methods

### Ethics

This study protocol was approved by the Institutional Review Board of Southwest Hospital, Third Military Medical University (No. 2016(94)), and registered on the clinical trial registration website (www.chictr.org.cn; Number: ChiCTR-IOR- 16,010,206) on 21 December 2016. Procedures were followed by the ethical standards of the committee on human experimentation and with the Helsinki Declaration of 1975. Written informed consent was obtained from all of the enrolled patients. All authors had access to the study data and had reviewed and approved the final manuscript.

### Patients’ Recruitment

**Fig. 1:**
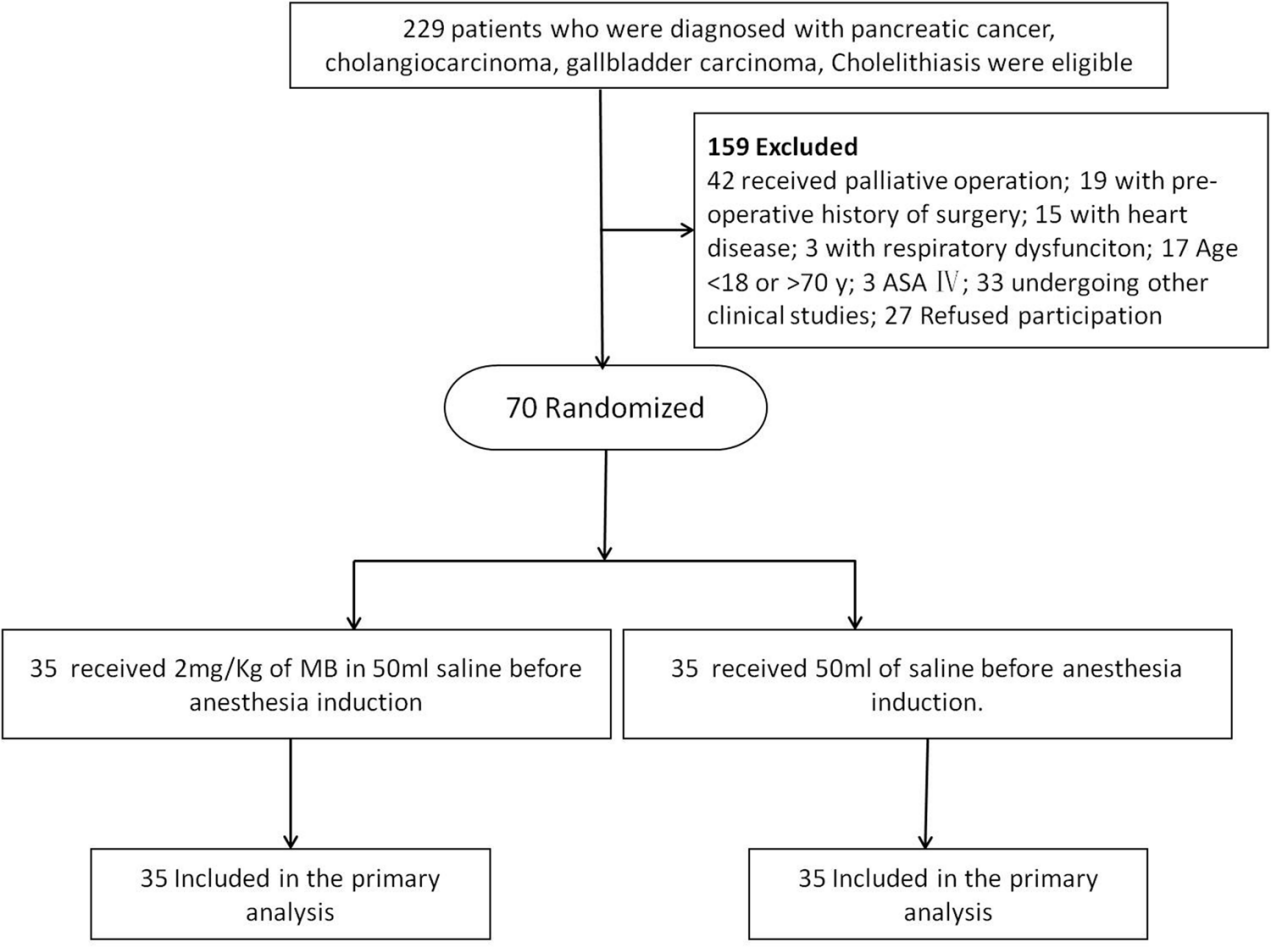
Flow chart of the trial

Patients with obstructive jaundice as indicated as TBIL > 5.0 mg/ml and the yellowing of skin or sclera, aged between 18 and 70 years old, who need surgical intervention, were enrolled in this study (Fig. 1) from January 15, 2017, to June 17, 2018, at Southwest hospital, the Third Military Medical University, Chongqing, China. Patients were excluded if they met any of the following criteria: anesthesiologists (ASA) physical status scores IV and V, the patients received selective serotonin reuptake inhibitor (SSRI) therapy, abdominal surgery history, coronary heart disease, hypertension, and any other cardiovascular diseases to be treated with medication, respiratory dysfunction including P_a_O_2_ ≤ 60 (F_i_O_2_ > 21%) or/and PaCO_2_ ≥ 50 mmHg, undergoing other clinical studies within recent 3 months or refusal to participate the study. The indications and operations performed for these patients are shown in the Supplemental Table 1 and Supplemental Table 2.

**Table 1:** Demographic characteristics and intra-operative details

	Control group (*n* = 35)	Methylene blue group (*n* = 35)	*P* value
Years	61 (51–65)	59(51–66)	0.624
Male/female	24/11	22/13	0.614
BMI	21.7 ± 2.9	22.3 ± 2.5	0.310
Diabetes	4	3	0.690
Smoking	14	15	0.808
ASA I/II/III	0/21/14	0/19/16	0.629
Etiology			
Cholangiocarcinoma	15	20	0.232
Gallbladder carcinoma	12	12	1.000
carcinoma of ampulla	5	2	0.232
Cholelithiasis	3	2	0.643
Pre-operatively ERCP	6/35	12/35	0.101
Total bilirubin (mM)	186.1(131.1–262.2)	212.9 (118.8–313.1)	0.364
Direct bilirubin (mM)	105.7 (95.1–116.4)	102.0 (68.5–178.5))	0.375
Indirect bilirubin (mM)	113.3 (71.4–133.4)	119.0 (62.4–130.7)	0.277
Bile acid (IU/L)	93.1 (16.3–188.9)	25.3 (9.7–138.9)	0.148
Creatinine (μmol)	61.5 (49.1–67.6)	63.4 (52.0–75.0)	0.812
Urea (mM)	4.4 (3.9–5.6)	4.6 (3.6–5.7)	0.935
glutamic oxalacetic transaminase(GOT) (IU/L)	137.5 (90.7–228.7)	101.1 (78.9–182.9)	0.351
glutamic-pyruvic transaminase (GPT) (IU/L)	154.4 (85.9–270.7)	129.6 (82.9–243.8)	0.582
Duration of anesthesia	8.8 ± 2.5	7.6 ± 2.2	0.033
Time of operation	7.6 ± 2.4	6.4 ± 2.0	0.029
Volume of blood loss (ml)	400 (300–500)	400 (200–450)	0.382
Volume of crystalloid solution (ml)	2200 (1600–2700)	2200 (1700–2600)	0.868
Volume of colloid solution (ml)	500 (325–1000)	500 (300–550)	0.188
Volume of washed red blood cells (ml)	0 (0–400)	0 (0–390)	0.170
Incidence of washed red blood cells infused	15	10	0.212
Volume of plasma (ml)	270 (0–380)	270 (0–300)	0.120
Incidence of plasma infused	19	10	0.029
Incidence of blood platelet infused	0	0	1.000
Unit of cryoprecipitate infused (U)	0 (0–0)	0 (0–0)	1.000
Incidence of cryoprecipitate infused	1	0	0.313
Volume of urine (ml)	1000 (600–1450)	1000 (600–1200)	0.301

**Table 2:** Vasopressor administration and anesthesia managements during operation

	Control group (*n* = 35)	Methylene blue group (*n* = 35)		*P* value
The incidence of norepinephrine used	63% (23/35)	37% (13/35)	0.017	
The incidence of total dose of norepinephrine used > 2 mg	26% (9/35)	3% (1/35)	0.006	
The dose of dobutamine used (mg)	0(0–0)	0(0–0)	0.513	
The incidence of dobutamine used	3% (1/35)	9% (3/35)	0.303	
Volume of washed red blood cells used (ml)	262.29 ± 359.96	154.86 ± 265.69	0.170	
Incidence of washed red blood cells infused	43% (15/35)	29% (10/35)	0.212	
Volume of plasma(ml)	228.00 ± 238.50	139.14 ± 222.66	0.120	
Incidence of plasma infused	54% (19/35)	29% (10/35)	0.029	
Volume of crystalloid solution infused (ml)	2207.43 ± 905.70	2241.14 ± 730.30	0.868	
Volume of colloidal solution infused (ml)	664.29 ± 505.74	517.14 ± 390.23	0.188	
Volume of blood loss (ml)	438.86 ± 302.39	378.29 ± 259.07	0.382	
Volume of urine(ml)	1096 ± 547.46	956.86 ± 517.41	0.301	
Duration of anesthesia (hrs)	8.79 ± 2.47	7.56 ± 2.13 (1.16)	0.033	
Duration of surgical procedure(hrs)	7.63 ± 2.31	6.44 ± 2.01 (1.18)	0.029	
ICU stay (day)	3.4 ± 1.4	2.5 ± 1.3	0.013	
Hospital stay (day)	18(14–23)	17 (13–27)	0.681	
AKI (KDIGO criteria)	23% (8/35)	6% (2/35)	0.040	
Six month’s mortality	11% (4/35)	9% (3/35)	0.690	

### Randomization

Randomization in a 1:1 ratio was done by an independent staff and all recruited patients were randomly allocated to receive either 2 mg/kg of Methylene blue I.V. in 50 ml of saline solution or 50 ml of saline solution. The researchers who collected data were blinded to the trial protocol, and the anesthesiologists who performed the anesthesia were blinded for grouping.

### Procedures

The dose of 2 mg/kg of the bodyweight of methylene blue (20 mg/2 ml) (Jichuan Medicine Co., Ltd, Taixing, China) was diluted with saline solution to be 50 ml; the placebo was 50 ml of saline solution. Both were prepared by a medical staff who did not participate in the rest study. Before general anesthesia induction, all patients received placement of a central venous catheter (B. Braun, Melsongen, Germany) and Swan-Ganz catheter via the right jugular vein, and an arterial catheter (18G) at the site of the left radial artery under local anesthesia. Invasive arterial blood pressure (ABP) and central venous pressure (CVP) were monitored continuously during operation. The surgical procedures were well matched between the two groups.

The methylene blue solution or normal saline was administrated by a continuous intravenous infusion at a rate of 2.5 ml/min before anesthesia induction, and then all the patients were induced with the administration of midazolam (0.02–0.05 mg/kg), sufentanil (0.5–1 μg/kg), etomidate (0.2–0.3 mg/kg), and cisatracurium (0.2–0.3 mg/kg). The patients received trachea intubation and ventilated with 8–10 ml/kg of tidal volume, 12–16 rpm, 1:2 of I:E to maintain E_t_CO_2_ between 35 and 40 cmH_2_O. Sevoflurane (1–2%) and continuous intravenous infusion of remifentanil at dose of 2–4 ng/ml of plasma concentration were administrated to maintain enough depth of anesthesia (40–60 of Bispectral Index (BIS)) throughout the surgical procedure. Cisatracurium (a bolus dose of 0.1–0.2 mg/kg) was injected when necessary to keep a single or zero twitch on the train-of-four stimulation of the ulnar nerve (TOF-Watch SX; Organon Ltd., Dublin, Ireland) during operation. The anesthesia protocol was identified for all patients. Arterial blood gas analysis was determined before and after anesthesia induction, after removal of the tumor, during the phase of entero-anastomosis or choledochojejunostomy, before closure of enterocele, and 24 h after the surgery completion.Hemodynamic status and blood transfusion was managed according to the Infusion Regimen (Supplemental Tables 3 and 4). Decisions regarding all other aspects of medical care during the intraoperative and postoperative periods, including pain management after operation, and administration of fluids and antibiotics, were made by attending surgeons according the routine clinical practices.

**Table 3:** Biochemical indexes of kidney and liver function

indexes	Time points	Control group (*n* = 35)	Methylene blue (*n* = 35)	*P* value
Creatinine (µmol)	Baseline	62.6 ± 18.7	63.6 ± 15.9	0.812
	The incidence of abnormal creatinine (%/*n*)	9% (3)	9% (3)	1.000
	1st day after operation	70.3 ± 25.4	69.6 ± 30.9	0.920
	The incidence of abnormal creatinine	26% (9)	20% (7)	0.570
	2nd day after Operation	63.3 ± 28.7	63.6 ± 47.9	0.972
	The incidence of abnormal creatinine	17% (6)	3% (1)	0.046
	3rd day after operation	57.9 ± 21	53.3 ± 12.8	0.308
	The incidence of abnormal creatinine	14% (5)	0 (0)	0.020
Glutamic oxalacetic transaminase(GOT) (IU/L)	Baseline	217.1 ± 228.1	168.4 ± 194.6	0.351
	The incidence of abnormal baseline GOT (%/n)	89% (31)	97% (34)	0.164
	1st day after operation	559.0 ± 837.6	255.2 ± 250.3	0.043
	2nd day after operation	297.7 ± 398.4	132.2 ± 138.9	0.029
	3rd day after operation	124.9 ± 136.9	71.0 ± 62.7	0.049
Glutamic-pyruvic transaminase (GPT) (IU/L)	Baseline	218 ± 229.1	191.3 ± 159.7	0.582
	The incidence of abnormal GPT	94% (33)	97% (34)	0.555
	1st day after operation	464.8 ± 583.3	241.2 ± 198.4	0.043
	2nd day after operation	344.5 ± 473.5	164.9 ± 156.1	0.044
	3rd day after operation	215.5 ± 289.6	117.2 ± 111.2	0.081
Creatine kinase (CK, IU/L)	1st day after operation	782.6 ± 380.4	441.1 ± 282.3	0.037
	2nd day after operation	569.5 ± 257.6	345.4 ± 276.5	0.018
	3rd day after operation	389.3 ± 198.6	177.2 ± 135.7	0.011

### Outcomes

The primary outcome was the frequency of norepinephrine administration during the operation phase. Secondary outcomes included the dose of norepinephrine, other vasoactive agent usage, the loading volume of fluid, liver and renal function, ICU stay, and hospital stay.

### Power Calculation and Statistical Analysis

Normally distributed data are reported as mean ± standard deviation, and abnormally distributed data are reported as medians and interquartile ranges. The D’Agostino-Pearson test was applied to assess data normality. Normally distributed variables were compared using the Student *t* test or paired Student *t*-test, as appropriate, and abnormally distributed data were compared by using the Mann–Whitney test. Categorical variables were compared using Fisher’s exact. (SPSS 20.0 software, Chicago, IL, USA). A *P*-value less than 0.05 was considered to be of statistical significance.

The frequency of noradrenaline administration to maintain mean blood pressure above 65 mmHg or > 80% of baseline during operation was about 70% in this comparable patient population in the previous study. If the frequency of noradrenaline administration during operation was reduced by half with the administration of methylene blue, then with a significance set at 0.05 and power set at 80%, the sample size required to detect differences was 66 patients in total for two arms. Taking into account a lost-to-follow-up rate of about 10%, 70 patients were enrolled.

## Results

Between January 15, 2017, and June 17, 2018, 229 patients were screened for study participation. Of those, 70 patients who met recruitment criteria were recruited and randomly assigned to receive either methylene blue or control (*n* = 35/group) (Fig. 1). During the study period, there were no lapses in the blinding. All patients were included for data analysis. The final visit of the last randomized patient was done on September 20, 2019. Overall, the patients in the control and methylene blue group were well matched for the baseline characteristics (Table 1).

### Vasopressor Administration During Operation

**Fig. 2:**
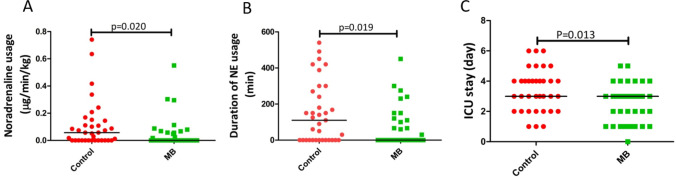
Noradrenaline (NE) usage and ICU stay. **A** Noradrenaline usage during operation_,_ **B** duration of NE administration, **C** duration of ICU stay. Data are presented as dot plots and mean ± S.E.M (*n* = 35)

Noradrenaline was administration to maintain MBP over 65 mmHg or > 80% of baseline, and SVR over 800 dyne/s/cm^5^. Less patients received noradrenaline in the methylene blue group than in the control group (13/35 vs 23/35, *P* = 0.017) (Table 2). The time-averaged dose of noradrenaline administration during operation was decreased in the methylene blue group than in the control group [(0.00 (0.00–0.06) μg/min/kg vs 0.06 (0.00–0.15) μg/min/kg, *P* = 0.020] (Fig. 2A). Less patients were received large dose of noradrenaline (total dose > 2 mg/throughout the procedure) infusion in the methylene blue group than in the control group (1/35 vs 9/35, *P* = 0.006) (Table 2). The duration of noradrenaline administration was decreased in the methylene blue compared with the control group [0(0–110) min vs 110(0–270), *P* = 0.019] (Fig. 2B). The frequency and dose of dobutamine administration during operation did not differ between the two groups (Table 2).

### Hemodynamic Parameters During Operation

**Fig. 3:**
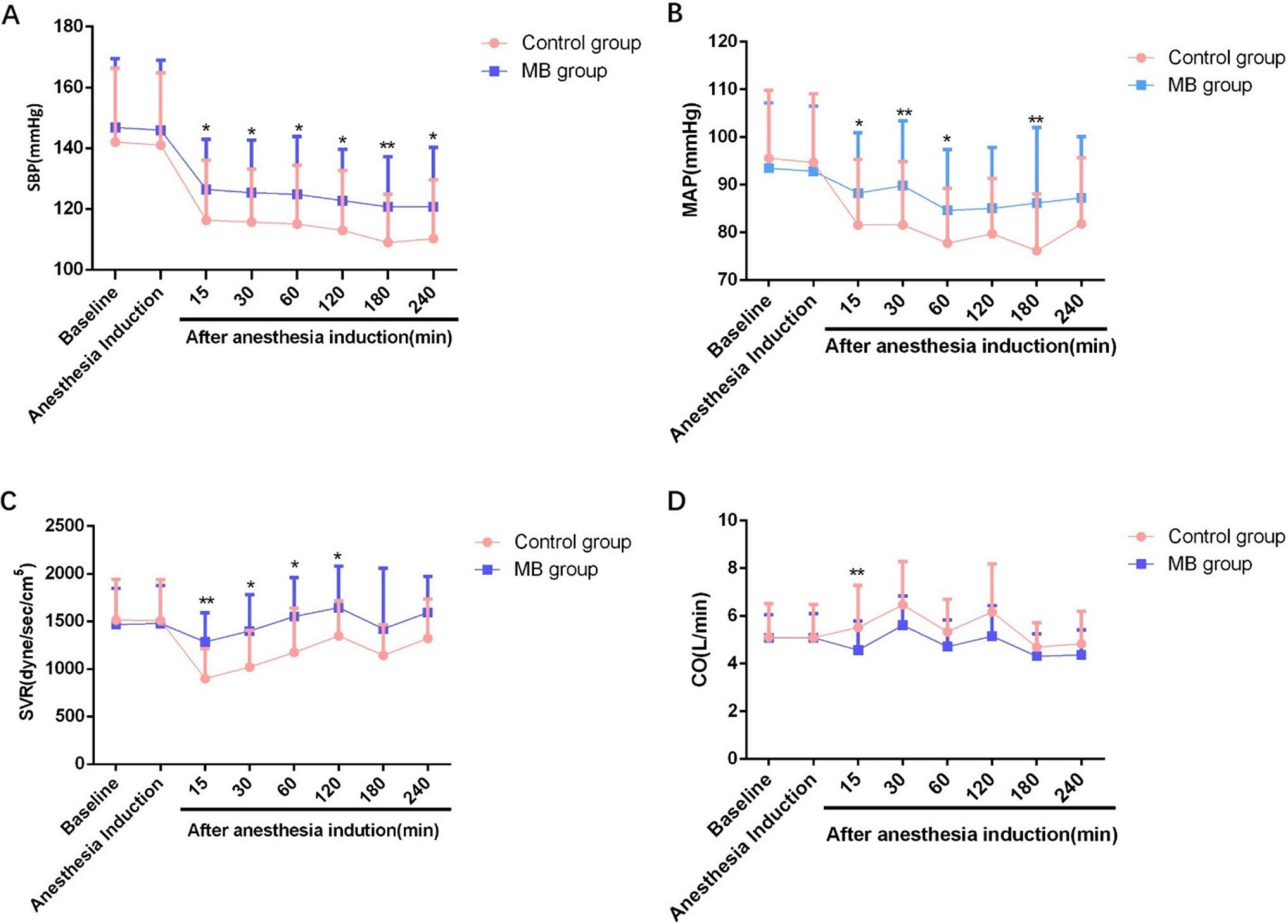
Hemodynamic parameters during operation. **A** SBP, **B** MAP, **C** SVR, and **D** CO during operation. Data are presented as dot plots and mean ± S.E.M (*n* = 35)

The baseline of SBP, MAP, SVR, and CO did not differ between the two groups. After anesthesia induction, SBP, MAP, and SVR were reduced in both groups. SBP (Fig. 3A) and MAP (Fig. 3B) were higher in the methylene blue group compared with the control group throughout the surgical procedure (*P* < 0.05). SVR was also higher in the methylene blue group compared with the control group until 180 min after anesthesia induction (*P* < 0.05) (Fig. 3C). CO was higher in the control group than the methylene blue group at 15 min after anesthesia induction (*P* < 0.05) and it was also higher in the control group than in the methylene blue group at other time points, but there was no significant difference (*P* > 0.05) (Fig. 3D).

### The Other Anesthesia Managements During the Operation

Less patients received plasma transfusion in the methylene blue group compared with the control group (10/35 *vs* 19/35, *P* = 0.029). The frequency of plasma infusion was reduced in the methylene blue group when compared with the control group (29% vs 43%, *P* = 0.029). There is no statistical difference of blood loss, washed red blood cells, colloid, crystalloid solution, and other blood products during operation. The duration of anesthesia (7.56 ± 2.13 h vs 8.79 ± 2.47 h, *P* = 0.033) and surgical procedure duration (6.44 ± 2.01 h vs 7.63 ± 2.31 h, *P* = 0.029) was reduced in the methylene blue group when compared with the control group, respectively (Table 2).

### Arterial Blood Gas Analysis

There was no significant difference of pH, Hb, PO_2_, PCO_2_, K^+^, Ca^2+^, BE, and lactic acid between the two groups at all the observed time points (Supplemental Table 5).

### Kidney and Liver Function

Fewer patients suffered from acute kidney injury after operation (KDIGO criteria) in the methylene blue group than in the control group (6% vs 23%, *P* = 0.040). Fewer patients in the methylene blue group presented an increase of creatinine over the normal range compared with the control group on the postoperative day 2 (3% vs 17%, *P* = 0.046) and day 3 (0 vs 14%, *P* = 0.020) (Table 3). 

Most of patients in the control and methylene blue group presented abnormally liver function, which was demonstrated the frequency of glutamic oxalacetic transaminase (GOT) over than (89% vs 97%, *P* = 0.164) and glutamic pyruvic transaminase (GPT) (94% vs 97%, *P* = 0.555) over normal range, respectively. GOT was increased at day 1st after the operation, then gradually declined over time, GOT was decreased in the methylene blue group compared with the control group on day 1st (255.2 ± 250.3 IU/L *vs* 559.0 ± 837.6 IU/L, *P* = 0.043), 2nd (132.2 ± 138.9 IU/L vs 297.7 ± 398.4 IU/L, *P* = 0.029), and 3rd (71.0 ± 62.7 IU/L vs 124.9 ± 136.9 IU/L, *P* = 0.049) after the surgery, respectively. GPT was increased on the 1st day after the surgery and then gradually declined over time. GPT a was decreased in methylene blue group compared with the control group at day 1st (241.2 ± 198.4 IU/L vs 464.8 ± 583.3 IU/L, *P* = 0.043), 2nd (164.9 ± 156.1 IU/L vs 344.5 ± 473.5 IU/L, *P* = 0.044), 3rd (117.2 ± 111.2 IU/L vs 215.5 ± 289.6 IU/L, *P* = 0.081) after the surgery, respectively. (Table 3)Creatine kinase (CK) was enhanced after surgery and declined gradually over time. CK activity was reduced in the methylene blue group compared with the control group on day 1st (441.1 ± 282.3 IU/L vs 782.6 ± 480.4 IU/L, *P* = 0.037), 2nd (345.4 ± 276.5 IU/L vs 569.5 ± 257.6 IU/L, *P* = 0.018), 3rd (177.2 ± 135.7 IU/L vs 389.3 ± 198.6 IU/L, *P* = 0.011) after operation, respectively (Table 3).

Other laboratory measurements did not differ between the two groups (Supplemental Table 6).

## Discussion

The current study suggests that prophylactic administration of methylene blue helps to maintain hemodynamic stabilization during procedures of relieving obstructive jaundice and thus improves renal and hepatic function, attenuating cardiac ischemic injury the after the operation, and shortened the ICU stay.

Patients with obstructive jaundice are at high risk for impairment of cardiovascular and low vascular response under anesthesia conditions and often need high dose of vasopressors to maintain stabilization of hemodynamic. It has been reported that a high requirement for vasoactive drugs is associated with increased mortality and organ’s function, which is due to that excessive vasoconstriction might be deleterious to micro-circulation and lead to mis-match between macro-circulation and micro-circulation during shock conditions.^12–14^ In a previous study, we found that reduced SVR associated obstructive jaundice resulted in this pronouncing hypotension, which at least partly attributed to this high morbidity and mortality after the operations.^15–17^ Increased bilirubin and bile acid and associated toxins were reported to contribute to this reduced vascular response.^18–20^ Nitric oxide (NO) was found to be increased in the patients with obstructive jaundice and played important roles in the vascular hypo-response and organs’ dysfunction in the patients with obstructive jaundice.^21^

In clinic practice, noradrenaline is a first-line drug to correct hypotension associated with reduced SVR^15,22^; however, this hypotension associated with obstructive jaundice does not often respond well to norepinephrine, and hence, a large dose of norepinephrine is often administrated to combat hypotension. It was found that microcirculatory is still abnormal despite normalization of blood pressure by vasopressors under this condition, which is considered the primary cause of kidney and hepatic dysfunction, and cardiac injury after the operations.^23^

Methylene blue has been suggested as an adjuvant treatment in the setting of refractory hypotension.^24,25^ Methylene blue reduces nitric oxide–mediated vasodilation via direct inhibitory of iNOS and soluble guanylate cyclase,^26^ which attenuates remarkably low response to vasoconstrictor. Methylene blue is recommended as an effective treatment of refractory hypotension which is not sensitive to catecholamine in the international guidelines.^27^ Recently, it has been reported that methylene blue improved both macro-circulation and micro-circulation, and thus attenuates mismatch between macro-circulation and micro-circulation induced by administration with high dose of catecholamine,^23^ which was regarded to contribute to improve hepatic and kidney function, and attenuated myocardium ischemic damage. It has been reported that methylene blue is routinely administrated as rescue therapy for catecholamine-refractory hypotension in the setting of cardiac surgery, sepsis shock, and liver transplantation. Moreover, it has been found that early administration is more effective than later administration in these reports.^11,22^ To our best knowledge, this clinical study firstly reported the protective effect of prophylactic administration of methylene blue in patients with obstruction jaundice. Prophylactic administration of methylene blue prevented occurrence of refractory hypotension during the operation and thus protected the patients from hepatic, kidney, and cardiac injury. These data suggest that the patients with obstructive jaundice benefit from prophylactic administration of methylene blue.

Some limitations of this study are the following. Firstly, this is single-center study with small sample size. Second, methylene blue was administrated before anesthesia induction; the risk and benefit of the patients with low risk of refractory hypotension remain unknown. Thirdly, the effects of methylene blue on long-term outcomes are also unknown and warrant further study. Fourthly, methylene blue may interfere some measurements, including pulse oximetry reading and alter the color of body fluid, such as urine; all these need to be closely monitored using its use and more cautions are needed to take. Lastly, some of the patients underwent pre-operation ERCP in both groups, because the number of patients who received pre-operation ERCP was not big enough to stratified analyze the effects of methylene blue and pre-operation ERCP.

Although owing to mentioned limitations, methylene blue may be recommended to be used to improve hemodynamic stabilization during operation and organ’ function in the patients undergoing operations relieving obstructive jaundice, but further clinical study is still needed to verify its long-term benefits.

## Conclusion

This study shows that prophylactic administration of methylene blue improves hemodynamic stabilization, and organs’ function via maintaining tissue perfusion in the patients undergoing the procedures of relieving obstructive jaundice. Patients with high risk of refractory hypotension may benefit from prophylactic administration of methylene blue but warrant further study.

### Supplementary Information

Below is the link to the electronic supplementary material.

Supplementary file1 (DOCX 21 kb)

Supplementary file2 (DOCX 20 kb)

Supplementary file3 (DOCX 19 kb)

Supplementary file4 (DOCX 18 kb)

Supplementary file5 (DOCX 26 kb)

Supplementary file6 (DOCX 27 kb)
