# Supplementary material for: Prophylactic Administration with Methylene Blue Improves Hemodynamic Stabilization During Obstructive Jaundice–Related Diseases’ Operation: a Blinded Randomized Controlled Trial
Source: J Gastrointest Surg. 2023 Apr 26;27(9):1837–45. doi: 10.1007/s11605-022-05499-3 (PMC10511601; doi:10.1007/s11605-022-05499-3)
Supplement: Supplementary file 1 — Supplementary file1 (DOCX 21 kb) [file 11605_2022_5499_MOESM1_ESM.docx]

Supplemental Table 1 etiology of obstructive jaundice

|  | Control group (n=35) | Methylene blue group(n=35) |
| --- | --- | --- |
| Adenocarcinoma of the ampulla of Vater | 1 | 0 |
| Cholangiocarcinoma | 3 | 1 |
| Chronic hilar cholangitis | 0 | 1 |
| Chronic pancreatitis and pancreatic duct stones | 1 | 0 |
| Duodenal adenocarcinoma | 0 | 2 |
| Duodenal papilla adenocarcinoma | 2 | 2 |
| Gallbladder carcinoma | 3 | 1 |
| Hepatocellular carcinoma | 0 | 1 |
| Hepatocholangiolithiasis | 1 | 0 |
| Hilar biliary stricture | 0 | 1 |
| Hilar cholangiocarcinoma | 6 | 4 |
| Lower bile duct adenocarcinoma | 1 | 2 |
| Lower choledochal carcinoma | 0 | 3 |
| Lower choledochal stenosis | 1 | 0 |
| Mass type pancreatitis | 0 | 1 |
| Pancreatic carcinoma | 5 | 7 |
| Pancreatic head carcinoma | 8 | 6 |
| Periampullary adenocarcinoma | 2 | 2 |
| recurrent Hepatocellular carcinoma | 1 | 1 |
