# Supplementary material for: Prophylactic Administration with Methylene Blue Improves Hemodynamic Stabilization During Obstructive Jaundice–Related Diseases’ Operation: a Blinded Randomized Controlled Trial
Source: J Gastrointest Surg. 2023 Apr 26;27(9):1837–45. doi: 10.1007/s11605-022-05499-3 (PMC10511601; doi:10.1007/s11605-022-05499-3)
Supplement: Supplementary file 2 — Supplementary file2 (DOCX 20 kb) [file 11605_2022_5499_MOESM2_ESM.docx]

Supplemental Table 2 Operations performed of obstructive jaundice related diseases' patients

| Type of procedures | Control group (n=35) | Methylene blue group (n=35) |  |
| --- | --- | --- | --- |
| Palliative operation | 6 | 11 |  |
| radical operation | 29 | 24 |  |
