# Supplementary material for: Prophylactic Administration with Methylene Blue Improves Hemodynamic Stabilization During Obstructive Jaundice–Related Diseases’ Operation: a Blinded Randomized Controlled Trial
Source: J Gastrointest Surg. 2023 Apr 26;27(9):1837–45. doi: 10.1007/s11605-022-05499-3 (PMC10511601; doi:10.1007/s11605-022-05499-3)
Supplement: Supplementary file 3 — Supplementary file3 (DOCX 19 kb) [file 11605_2022_5499_MOESM3_ESM.docx]

Supplemental Table 3 Infusion Regimen

| Mean Blood pressure | SVR (dyne/sec/cm^5^) | CO  (L/min) | management |
| --- | --- | --- | --- |
| <65 mmHg or decrease  to 80% of baseline value | <800 | >4 | Infusion of norepinephrine  at start of 40 ng/kg/min |
| <65 mmHg or decrease  to 80% of baseline value | >800 | <4 | Infusion of dobutamine  at start of 5 ng/kg/min |
| <65 mmHg or decrease  to 80% of baseline value | <800 | <4 | Infusion of both norepinephrine and  dobutamine |
