# Supplementary material for: Prophylactic Administration with Methylene Blue Improves Hemodynamic Stabilization During Obstructive Jaundice–Related Diseases’ Operation: a Blinded Randomized Controlled Trial
Source: J Gastrointest Surg. 2023 Apr 26;27(9):1837–45. doi: 10.1007/s11605-022-05499-3 (PMC10511601; doi:10.1007/s11605-022-05499-3)
Supplement: Supplementary file 4 — Supplementary file4 (DOCX 18 kb) [file 11605_2022_5499_MOESM4_ESM.docx]

Supplemental Table 4 Infusion Regimen of blood products

| Hb | Hemodynamic | management |
| --- | --- | --- |
| <7 g/dl | ~ | Infusion of washed red cell |
| >7 g/dl and <10 g/dl | instability | Infusion of washed red cell |
