# Supplementary material for: Prophylactic Administration with Methylene Blue Improves Hemodynamic Stabilization During Obstructive Jaundice–Related Diseases’ Operation: a Blinded Randomized Controlled Trial
Source: J Gastrointest Surg. 2023 Apr 26;27(9):1837–45. doi: 10.1007/s11605-022-05499-3 (PMC10511601; doi:10.1007/s11605-022-05499-3)
Supplement: Supplementary file 5 — Supplementary file5 (DOCX 26 kb) [file 11605_2022_5499_MOESM5_ESM.docx]

Supplemental Table 5 Arterial blood gas analysis

| Time points | Indexes | Control group (n=35) | Methylene blue group (n=35) | *P* value |
| --- | --- | --- | --- | --- |
| Prior to Anesthesia Induction | PH | 7.39±0.03 | 7.4±0.04 | 0.307 |
|  | Hb | 115±17.18 | 119.46±16.77 | 0.283 |
|  | PO_2_(60%) | 87.29±40.17 | 80.36±18.19 | 0.364 |
|  | PCO_2_ | 38.24±4.26 | 37.18±4.27 | 0.311 |
|  | K^+^ | 3.44±0.43 | 3.39±0.4 | 0.592 |
|  | Ca^2+^ | 1.2±0.05 | 1.19±0.07 | 0.804 |
|  | BE | -1.52±2.11 | -1.51±2.23 | 0.991 |
|  | LAC | 1.01±0.38 | 1.1±0.54 | 0.400 |
| 30min after Anesthesia Induction | PH | 7.39±0.05 | 7.38±0.05 | 0.388 |
|  | Hb | 104.94±16.65 | 107.37±15.19 | 0.532 |
|  | PO_2_(60%) | 247.74±66.56 | 232.53±50.11 | 0.305 |
|  | PCO_2_ | 37.41±3.6 | 38.27±4.7 | 0.400 |
|  | K^+^ | 3.52±0.55 | 3.49±0.45 | 0.816 |
|  | Ca^2+^ | 1.19±0.06 | 1.19±0.06 | 0.985 |
|  | BE | -1.94±2.32 | -2.35±2.24 | 0.453 |
|  | LAC | 0.9±0.71 | 0.95±0.64 | 0.769 |
| After removal of tumor mass | PH | 7.35±0.05 | 7.35±0.06 | 0.941 |
|  | Hb | 106.79±14.96 | 107±15.8 | 0.956 |
|  | PO_2_(60%) | 239.3±54.46 | 222.76±47.07 | 0.198 |
|  | PCO_2_ | 39.84±4.56 | 39.61±6.06 | 0.864 |
|  | K^+^ | 3.63±0.49 | 3.57±0.47 | 0.652 |
|  | Ca^2+^ | 1.15±0.07 | 1.16±0.05 | 0.538 |
|  | BE | -3.24±2.4 | -3.32±2.53 | 0.891 |
|  | LAC | 1.4±0.47 | 1.48±0.83 | 0.629 |
| During anastomosis | PH | 7.35±0.05 | 7.36±0.05 | 0.576 |
|  | Hb | 101.03±14.61 | 105.85±16.47 | 0.213 |
|  | PO_2_(60%) | 250.44±57.25 | 224.73±46.87 | 0.052 |
|  | PCO_2_ | 39.24±3.92 | 40.37±5.46 | 0.339 |
|  | K^+^ | 3.69±0.55 | 3.58±0.41 | 0.330 |
|  | Ca^2+^ | 1.14±0.06 | 1.13±0.06 | 0.387 |
|  | BE | -3.4±2.48 | -2.53±2.28 | 0.140 |
|  | LAC | 1.61±0.73 | 1.46±0.86 | 0.452 |
| Before closure of enterocoelia | PH | 7.35±0.05 | 7.35±0.05 | 0.939 |
|  | Hb | 104.53±14.11 | 107.91±11.54 | 0.295 |
|  | PO_2_(60%) | 245.09±75.59 | 219.8±56.27 | 0.134 |
|  | PCO_2_ | 39.45±3.81 | 40.63±5.6 | 0.321 |
|  | K^+^ | 3.76±0.55 | 3.61±0.36 | 0.195 |
|  | Ca^2+^ | 1.13±0.06 | 1.12±0.06 | 0.468 |
|  | BE | -3.49±2.58 | -3.05±1.92 | 0.436 |
|  | LAC | 1.73±0.87 | 1.63±0.95 | 0.663 |
| 24 hrs after operation | PH | 7.38±0.03 | 7.35±0.04 | 0.147 |
|  | Hb | 111.3±21.3 | 95.1±30.83 | 0.211 |
|  | PO_2_(60%) | 193.27±75.05 | 167.04±53.04 | 0.403 |
|  | PCO_2_ | 40.31±3 | 41.97±4.21 | 0.348 |
|  | K^+^ | 3.54±0.47 | 3.57±0.27 | 0.870 |
|  | Ca^2+^ | 1.15±0.05 | 1.11±0.02 | 0.095 |
|  | BE | -2.12±3.15 | -1.35±2.30 | 0.558 |
|  | LAC | 2.51±2.04 | 2.22±0.57 | 0.650 |

LAC: lactic acid
