# Supplementary material for: Prophylactic Administration with Methylene Blue Improves Hemodynamic Stabilization During Obstructive Jaundice–Related Diseases’ Operation: a Blinded Randomized Controlled Trial
Source: J Gastrointest Surg. 2023 Apr 26;27(9):1837–45. doi: 10.1007/s11605-022-05499-3 (PMC10511601; doi:10.1007/s11605-022-05499-3)
Supplement: Supplementary file 6 — Supplementary file6 (DOCX 27 kb) [file 11605_2022_5499_MOESM6_ESM.docx]

Supplemental Table 6 Other laboratory measurements of biomarkers' level

| Biomarkers | Time points | Control group (n=35) | Methylene blue group (n=35) | *P* value |
| --- | --- | --- | --- | --- |
| Urean nitrogen（UN, mmol） | Baseline prior to Anesthesia induction | 4.8±1.5 | 4.8±1.6 | 0.935 |
|  | 1^st^ day after Anesthesia induction | 5.6±2.2 | 5.5±2.5 | 0.744 |
|  | 2^nd^ day after Anesthesia induction | 7±3.2 | 5.9±2.5 | 0.144 |
|  | 3^rd^ day after Anesthesia induction | 7±3.3 | 6.3±1.9 | 0.309 |
| estimated glomerular filtration rate  (eGFR) | Baseline prior to Anesthesia induction | 103.6±13.4 | 102.3±14.4 | 0.718 |
|  | 1^st^ day after Anesthesia induction | 94±21.4 | 98.4±19.1 | 0.391 |
|  | 2^nd^ day after Anesthesia induction | 100.6±22 | 107.1±12.4 | 0.160 |
|  | 3^rd^ day after Anesthesia induction | 104.4±18.3 | 108.8±13.7 | 0.290 |
| β-microglobulin (mg/L) | Baseline prior to Anesthesia induction | 2±1 | 2.2±0.9 | 0.511 |
|  | 1^st^ day after Anesthesia induction | 2.1±1.2 | 2.3±1.1 | 0.427 |
|  | 2^nd^ day after Anesthesia induction | 2.1±1.1 | 2.2±0.9 | 0.608 |
|  | 3^rd^ day after Anesthesia induction | 2.2±0.9 | 2.4±0.9 | 0.291 |
| α- microglobulin(mg/L) | Baseline prior to Anesthesia induction | 20.7±9.6 | 17.2±4.2 | 0.063 |
|  | 1^st^ day after Anesthesia induction | 14.8±4.2 | 13.5±4.5 | 0.250 |
|  | 2^nd^ day after Anesthesia induction | 14.8±4.7 | 14.8±3.8 | 0.948 |
|  | 3^rd^ day after Anesthesia induction | 16±4.4 | 15.5±3.2 | 0.586 |
| alkaline phosphatase (ALP, IU/L) | Baseline prior to Anesthesia induction | 497.1±319.3 | 458.3±478 | 0.696 |
|  | 1^st^ day after Anesthesia induction | 337.1±188.2 | 349.3±422.8 | 0.878 |
|  | 2^nd^ day after Anesthesia induction | 252.1±123.9 | 256.4±299.4 | 0.938 |
|  | 3^rd^ day after Anesthesia induction | 217.8±106 | 209.1±188.5 | 0.819 |
| Glutamine transpeptidase (GTT, IU/L) | Baseline prior to Anesthesia induction | 700.5±562.9 | 662.4±519.6 | 0.775 |
|  | 1^st^ day after Anesthesia induction | 380.6±341.3 | 337±318.4 | 0.591 |
|  | 2^rd^ day after Anesthesia induction | 270.2±263.9 | 246.2±222.7 | 0.688 |
|  | 3^rd^ day after Anesthesia induction | 194.8±178.8 | 226±186.7 | 0.497 |
| Sialic acid (mmol/L) | Baseline prior to Anesthesia induction | 619.8±101.5 | 654.3±112.6 | 0.192 |
|  | 1^st^ day after Anesthesia induction | 530.1±97.6 | 535±125.4 | 0.856 |
|  | 2^nd^ day after Anesthesia induction | 609.3±132.9 | 618.1±131.2 | 0.787 |
|  | 3^rd^ day after Anesthesia induction | 663.7±116.5 | 719.2±121 | 0.066 |
| [cholinesterase](javascript:;) (U/L) | Baseline prior to Anesthesia induction | 5357±2173.2 | 5545.3±1676.8 | 0.693 |
|  | 1^st^ day after Anesthesia induction | 3749.5±1096.1 | 3989.9±1069.7 | 0.371 |
|  | 2^nd^ day after Anesthesia induction | 2999.4±867.9 | 3119.1±837.1 | 0.568 |
|  | 3^rd^ day after Anesthesia induction | 2713.7±770.5 | 3009.2±753.9 | 0.126 |
| Superoxide dismutase (U/L) | Baseline prior to Anesthesia induction | 119.8±25.8 | 132.8±31.4 | 0.069 |
|  | 1^st^ day after Anesthesia induction | 113±22.9 | 119.3±31.3 | 0.351 |
|  | 2^nd^ day after Anesthesia induction | 113.1±31.7 | 104.1±22.6 | 0.196 |
|  | 3^rd^ day after Anesthesia induction | 105.3±25.4 | 101±22.6 | 0.480 |
| Lactic dehydrogenase (U/L) | 1^st^ day after Anesthesia induction | 393.3±217 | 350.9±122.7 | 0.610 |
|  | 2^nd^ day after Anesthesia induction | 245.7±103.8 | 253.1±91.4 | 0.887 |
|  | 3^rd^ day after Anesthesia induction | 190.2±85.8 | 185.4±66.3 | 0.919 |
